# Functional ZnONPs‐modified biochar derived from *Funtumia elastica* husk as an efficient adsorbent for the removal of sulfamethoxazole from wastewater

**DOI:** 10.1007/s11356-024-35594-8

**Published:** 2024-11-25

**Authors:** James Friday Amaku, Fanyana M. Mtunzi

**Affiliations:** 1 Wastewater Treatment Research Laboratory, Department of Biotechnology and Chemistry, Vaal University of Technology, Vanderbijlpark, 1911 Gauteng South Africa; 2 Department of Chemistry, Michael Okpara University of Agriculture Umudike, P.M.B 7267, Umuahia, Abia State Nigeria

**Keywords:** Adsorption, Antioxidant, Antimicrobial, Biochar, Thermodynamics, *Funtumia elastica* husk

## Abstract

*Funtumia elastica* husk was employed as an efficient and economically viable adsorbent to supplement traditional treatment methods in the removal of sulfamethoxazole from wastewater by converting it into usable material. The purpose of this study was to make biochar (FHB) from *Funtumia elastica* husk through the pyrolysis process and further modify the biochar using zinc oxide nanoparticles (ZnONPs) to a nanocomposite (FBZC). The antioxidant and antimicrobial characteristics as well as the potential of FBZC and FHB to sequester sulfamethoxazole from wastewater were investigated. Uptake capacities of 59.34 mg g^−1^ and 26.18 mg g^−1^ were attained for the monolayer adsorption of SMX onto FBZC and FHB, respectively. SEM and FTIR spectroscopic techniques were used to determine the surface morphology and chemical moieties of adsorbents, respectively. Brunauer–Emmett–teller (BET) surface analysis was used to assess the specific surface area of FHB (0.5643 m^2^ g^−1^) and FBZC (1.2267 m^2^ g^−1^). The Elovich and pseudo-first-order models are both well-fitted by the experimental data for FHB and FBZC, according to kinetic results. Nonetheless, the equilibrium data for FHB and FBZC were better explained by the Freundlich and Langmuir isotherm models, respectively. The pH_PZC_ values of 6.83 and 5.57 were determined for FBZC and FHB respectively. Optimum solution pH, dosage, and contact time of 6, 0.05 g, and 120 min were estimated for FHB and FBZC. In conclusion, these findings demonstrate the strong potential of FBZC to simultaneously arrest the spread of pathogenic microbes and sequester sulfamethoxazole from wastewater.

**Supplementary information:**

The online version contains supplementary material available at 10.1007/s11356-024-35594-8.

## Introduction

In aquaculture, animal husbandry, and medicine, antibiotics are widely employed. They have also been synthesized extensively (Noor et al. 2023; Nazmara et al. 2022). This particular class of chemicals has been essential to maintaining a safe environment. Due to the vital role of antibiotics to man and its environment, over 20,000 tons are manufactured yearly (Tzeng et al. 2016) and 30–90% of these drugs enter either as metabolites or parent compounds into the environment through digestive/biosolids (Li et al. 2019; Yousefi et al. 2021), wastewater treatment plants (WWTPs) (Joss et al. 2005), livestock excreta (Tolls 2001), and aquaculture wastewater (Luo et al. 2011). The increased frequency of antibiotics introduction into the environment has posed a high risk of great concern and has attracted the attention of stakeholders (Singh et al. 2019). Notwithstanding the small concentrations of antibiotics (between ng dm^−3^ to low mg dm^−3^) detected in the effluent of different countries (Singh et al. 2019; Khan et al. 2021), the build-up of antibiotic residues in the environment could lead to pathogenic bacteria developing resistance, making disease treatment more challenging and the imbalance of microbial ecosystems (Reguyal and Sarmah 2018b).

A range of treatment procedures have been used to remove medications that are subject to stakeholder monitoring and regulation (Oskoei et al. 2016). These techniques include advanced oxidation (Qi et al. 2020; Wang and Wang 2018), biodegradation (Liang DongHui and Hu YongYou 2019; Wang and Wang 2016), adsorption (Cheng et al. 2022), chemical oxidation (Liu et al. 2021a, b), photocatalytic degradation (Długosz et al. 2015; Wang and Zhuan 2020), and ozonation (Gonçalves et al. 2013; Wang and Chen 2020). Among the aforementioned treatment techniques, adsorption has been considered by many researchers to be the cheapest, easiest, and most efficient decontamination technique. Meanwhile, adsorbents such as multi-walled carbon nanotubes (Zhang et al. 2011), *Pithophora* sp. (Kumar et al. 2005), biochar (Shakya and Agarwal 2019; Dong et al. 2021), metal–organic framework (Wang et al. 2021), clay (Foroutan et al. 2020), seeds (Sen et al. 2016), microplastics (Zhang et al. 2020a, b), zeolite (Wu et al. 2010; Wang and Ariyanto 2007), nanocomposite (Sharifi et al. 2019), perlite (Govindasamy et al. 2009), groundnut shell (Bayuo et al. 2019), wheat bran (Wang et al. 2008), hydrogel (Vilela et al. 2019), and water hyacinth roots (Kumar and Chauhan 2019) among others have been employed for the elimination of contaminants from the aqueous phase.

Wang et al. synthesized a novel cobalt ferrite–loaded carbon nanotubes (CNTs/CoFe_2_O_4_) composite for the sequestration SMZ from aquatic ecosystems. The uptake potential of the composite was optimum in the acidic medium and the composite was easily thermally (300 ℃) regenerated for reuse (Wang et al. 2015). Cheng et al. reported the application of a metal–organic framework coated with imprinted polymer for swift and favourably selective adsorption of sulfamethoxazole from the aquatic ecosystem with an adsorption equilibrium time of 10 min, selectivity coefficient of 11.36, uptake potential of 284.66 mg g^−1^, and excellent reusability (Cheng et al. 2022). Activated carbon exhibits good removal efficiency with poor regeneration tendency. The high cost (1500–8900 per tonne) of activated carbon is a drawback to its application in environmental remediation practice (Klasson et al. 2009). Hence, it is imperative to fabricate cheap and effective adsorbents for the elimination of SMX from the aquatic environment. Biochar is a solid carbonaceous material that is made by pyrolyzing biomass, which includes sewage sludge, animal dung, and crop leftovers. The application of biochar in environmental remediation practice is becoming well-known because of its low-cost of production (US $246 per tonne) (McCarl et al. 2012). Several reports have demonstrated the capacity of biochar and biochar-based composites to effectively eliminate water contaminants (Cao et al. 2009; Liu and Zhang 2009; Yao et al. 2011; Sun et al. 2011).

However, the drawback to the utilization of biochar could be credited to the limited surface functional groups; hence, surface modification becomes essential to ensure adsorbate bias. Nanometal decorated biochar has shown enhanced removal capacity for different types of water contaminants (Sarojini et al. 2023; Choi et al. 2022; Huang et al. 2019; Yuan et al. 2020). Zinc oxide nanoparticles are metal oxides that are employed in different fields for the fabrication of unique materials. The exceptional qualities of ZnO-based materials may be attributed to their photocatalytic, electronic, and UV characteristics, and antimicrobial properties. On the other hand, ZnONPs are often employed in cosmetics production (Newman et al. 2009; Hatamie et al. 2015). This quality gives ZnO-based materials the superior edge in water treatment practice. Herein, we report primarily the fabrication of ZnO-coated biochar for the adsorption of SMX. The study aims at the design nanocomposite sustaining the capacity to disinfect and decontaminate aquatic ecosystem. The pristine and modified biochar were characterized using non-destructive spectroscopic techniques. The effects of temperature, pH, dose, and concentration on adsorption were evaluated. The thermodynamic parameters, equilibrium isotherms, and kinetics of the adsorptive removal of SMX by FHB and FBZC were investigated, and the outcome was compared with similar studies that employed different adsorbents.

## Materials and methods

Sulfamethoxazole (purity > 98%), sodium hydroxide (NaOH, 97%), hydrochloric acid (HCl, 36%), ascorbic acid (C_6_H_8_O_6_, 99.5%), nitric acid (HNO_3_, 98%), ethanol (99.9%), sodium chloride (NaCl, 99%), sulphuric acid (H_2_SO_4_, 98%), and acetone (99.9%) were acquired from Sigma-Aldrich and employed without additional processing.

### Preparation of stock solutions

SMX initial concentration was obtained by dissolving a known mass (1000 mg) of SMX in deionized water (1 dm^3^) in an appropriate standard volumetric flask and made up to the mark. Thereafter, the working concentration (100 mg dm^−3^) was obtained via a serial dilution of the stock solutions.

### Preparation of biomass

The dried husks of *Funtumia elastica* were gathered from the premises of the National Root Crops Research Institute, Umudike, Abia State, Nigeria. They were subsequently air-dried for 6 days. Following this, the dried samples were pulverized using an electric blender and then sieved through a 150-µm mesh screen. The processed biomass was then kept for future application in an airtight plastic bag.

### Preparation of biochar

The pulverized *Funtumia elastica* husk biowaste was pyrolyzed at a temperature of 350 °C for 90 min. Inside the tubular furnace, the pyrolysis process was performed with a limited air supply. The resulting biochar (FHB) was then washed using acetone, ethanol, deionized water, and oven-dried. The biochar was then ground and passed through a 100-mesh sieve. The fine black product was stored for future application.

### Nanocomposite fabrication (FBZC)

Biochar/ZnONPs nanocomposite were synthesized using biochar, aqueous solutions of zinc acetate dihydrate, and sodium hydroxide. This involved dissolving 22.8 g (0.124 mol) of zinc acetate dihydrate in 750 cm^3^ of deionized water containing 1 g of FHB. Another solution consisting of 6.0 g (0.1 moL) of NaOH in 1500 cm^3^ of deionized water was prepared. Both solutions were mixed by adding the aqueous NaOH dropwise under magnetic stirring and maintaining the stirring for 30 min. The mixture was then filtered and the black precipitates (FBZC) were repeatedly cleaned with 100% ethanol and distilled water. The resulting precipitates were dried at 105 °C, calcined for 30 min at 300 °C, and placed in a vacuum oven dryer for 24 h at 60 °C. About 1 g of ZnO-Biochar was transferred to 20 cm^3^ (0.01 M) of ascorbic acid and stirred to dryness at 60 ℃ to obtain FBZC.

### Characterization

Bruker D8 Advance powder x-ray diffraction (Bruker, US) was used to acquire the diffraction patterns of FBZC and FHB by making use of CuKa radiation (*k* = 1.54 nm) at a scan speed of 0.02/s. Surface area and pore characteristics of FBZC and FHB were investigated by making use of a Brunauer–Emmett–Teller (BET) analyzer from Micromeritics Instruments Corp, USA. Preceding the surface area measurements, FBZC and FHB were degassed at 110 ℃ for 24 h under vacuum to remove moisture. Successful fabrication of the adsorbents and the uptake of SMX onto the surface of FBZC and FHB was assessed using the Fourier transform infrared (FTIR) spectroscopy analyses (ThermoFisher Scientific, Waltham, MA, USA). Scanning electron microscopy (SEM) micrographs of FBZC and FHB were acquired using JSM-7500 F, JEOL, Tokyo, Japan.

### *Point of zero charge (pH*_*PZC*_*)*

In order to ascertain the pH at which the surface of FBZC and FHB will sustain a net charge of zero, approximately, 0.1 g of FBZC or FHB was added to eleven 250 cm^3^ conical flasks. Each flask held 50 cm^3^ of 0.1 mol dm^−3^ NaCl solution, adjusted from 2 to 12. The flasks were sealed and left to agitate in a pre-heated water bath that was kept at 25 °C for 48 h. After 48 h, the final pH of each mixture was determined. The pH_PZC_ of FBZC and FHB were then extrapolated from the line intercept of a graph that compares the initial and final pH (Mondal and Basu 2019).

### Antioxidant assay

The antioxidant characteristics of FBZC and FHB were assessed using the DPPH assay. Briefly, about 0.5 cm^3^ of DPPH (0.3 mM) solution was added to varied concentrations (25, 50, 100, 200, and 400 g cm^−1^) of FBZC or FHB. The wastewater treatment agent (FBZC and FHB) functioned as a radical scavenger, and DPPH was employed as a radical source. The mixtures of the radical scavenger and radical source were incubated in a dark compartment at 25 ℃ for 30 min. The concentration of the radical was estimated using the change in percentage of absorption wavelength at 517 nm (Begum et al. 2022). The inhibition of DPPH was calculated using Eq. 1.

1 $$I\%=\frac{\left({Asorbance}_{control}-{Asorbance}_{Sample}\right)}{{Asorbance}_{control}}\times 100$$

### Antibacterial activity

The agar well diffusion method was used to assess the antimicrobial characteristics of FBZC and FHB. The water treatment agents (FBZC and FHB) were evaluated against gram-positive (*Staphylococcus aureus*) and gram-negative (*Escherichia coli*) pathogenic bacteria in which ciprofloxacin was employed as positive control in the study. Microbial suspensions were inoculated with Muller-Hinton Agar Medium in Petri plates. About 250 µg of FBZC or FHB samples was contacted with the bacterial cultures in wells of approximately 10 mm in diameter bored with a well cutter. At 37 °C, the plates were incubated for 24 h and the antibacterial activity of FBZC and FHB was determined by measuring the zone of inhibition formed around the well (Qamar et al. 2017).

### Sorption experiments of SMX on FBZC and FHB

In the batch experiment, the optimum dosage for the uptake of SMX by FBZC or FHB was assessed by varying the adsorbent dose (0.01–0.1 g). The implication of solution pH, initial concentration, and contact time was examined from pH 2 to 10 at 298 K, 10–100 mg dm^−3^, and 5–180 min respectively. The effect of solution temperature on the adsorptive behaviour of FBZC and FHB was observed within 298–318 K. A standard working solution of SMX was freshly prepared from the stock solution and adjusted to the desired pH using either 0.1 mol dm^−3^ NaOH or 0.1 mol dm^−3^ HCl solutions. Approximately 50 mg of the adsorbents was introduced into stoppered amber glass bottles containing 25 cm^3^ of the SMX solution. The mixture underwent agitation for 180 min in a thermostated shaking water bath set at a fixed temperature of 298 K with an agitation speed of 150 rpm. Following agitation, the mixtures were filtered under gravity, and the residual concentration of SMX ions in the filtrate was determined using UV–visible spectrophotometry (Shimadzu UV-3600) at a wavelength of 268 nm. All experiments were performed in duplicate. Equations (2) and (3) were used to determine the adsorption capacity (mg g^−1^) and adsorption efficiency (% adsorbed) of FBZC and FHB, respectively:

2 $${q}_{eq}=\left(\frac{{C}_{i}-{C}_{eq}}{m}\right)V$$

3 $$\% adsorbed=\left(\frac{{C}_{i}-{C}_{eq}}{{C}_{i}}\right)\times 100$$

where *V* is the volume of the adsorbate. *C*_*i*_ is the initial adsorbate concentration (mg dm^−3^), *C*_*eq*_ is the equilibrium concentration (mg dm^−3^) of the adsorbate after adsorption, and *m* is the mass (g) of FBZC or FHB.

### Kinetics and isotherm models

Experimental data acquired from the contact time and initial concentration experiments were fitted into non-linear kinetic and isotherm models, respectively, to determine the effectiveness and mechanism responsible for SMX adsorption onto FBZC and FHB. Models of intraparticle diffusion, pseudo-second order, pseudo-first order, and Elovich kinetics were employed (see Table 1). On the other hand, the isotherm analysis was conducted using Freundlich and Langmuir (refer to Table 2).

**Table 1:** Kinetics models used to assess the uptake of SMX onto FBZC and FHB

Kinetic models	Equations	Parameters	References
Pseudo-first order	$\frac{{dq}_{t}}{{d}_{t}}={k}_{1}\left({q}_{e}-{q}_{t}\right)$	${q}_{e}{,k}_{1}$	Aksu and Karabayır (2008)
Pseudo-second order	$\frac{{dq}_{t}}{{d}_{t}}={k}_{2}{\left({q}_{e}-{q}_{t}\right)}^{2}$	${k}_{2},{q}_{e}$	Sevim et al. (2011)
Weber-Morris intraparticle diffusion	$\frac{{dq}_{t}}{{d}_{{t}^{-0.5}}}={k}_{id}$	${k}_{id,} l$	Ofomaja et al. (2009)
Elovich	$\frac{{dq}_{t}}{{d}_{t}}=\alpha exp\left(-\beta {q}_{t}\right)$	$\alpha , \beta$	Omorogie et al. (2016)

*α*, adsorption rate constant (mg g^−1^ min^−1^); *k*_*1*_, pseudo-first-order rate constant (min^−1^);. *k*_*id*_, intraparticle diffusion rate constant (mg g^−1^ min^0.5^); *q*_*e*_, quantity of adsorbate adsorbed at equilibrium (mg g^−1^); *l*, is a constant related to the boundary layer thickness (mg g^−1^); *k*_*2*_, pseudo-second-order rate constant (g mg^−1^ min^−1^) *q*_*t*_, quantity of adsorbate adsorbed at time t (mg g^−1^); *β*, desorption rate constant (g mg^−1^).

**Table 2:** Isotherm equations and parameters used to describe the uptake of SMX onto FBZC and FHB

Isotherm model	Equation	Parameters	References
Langmuir	${q}_{e}=\frac{{q}_{max}{bC}_{e}}{1+b{C}_{e}}$	${q}_{max},$ *b*	Langmuir (1918)
Freundlich	${q}_{e}={K}_{F}{C}_{e}^{{~}^{1}\!\left/ \!{~}_{n}\right.}$	${k}_{F}, n$	Freundlich (1906)

*q*_*eq*_, adsorption capacity (mg g^−1^) of FBZC and FHB; *C*_*eq*_, equilibrium concentration of SMX in solution (mg dm^−3^); *q*_*max*_, maximum monolayer potential (mg g^−1^) of FBZC and FHB; *b*, Langmuir isotherm constant (dm^3^ mg^−1^); *K*_*F*_, Freundlich isotherm constant (mg g^−1^) (dm^−3^ mg^−1^)^*n*^; *n*, adsorption intensity.

### Data analysis

Using the R statistical computing environments, the NLS non-linear regression procedure was used to fit the experimental data acquired from temperature, concentration, and time experiments into their respective models (Team R Core 2014). The adequacy of the models utilized in this investigation was verified by calculating and utilizing their residuals.

## Results and discussion

**Fig. 1:**
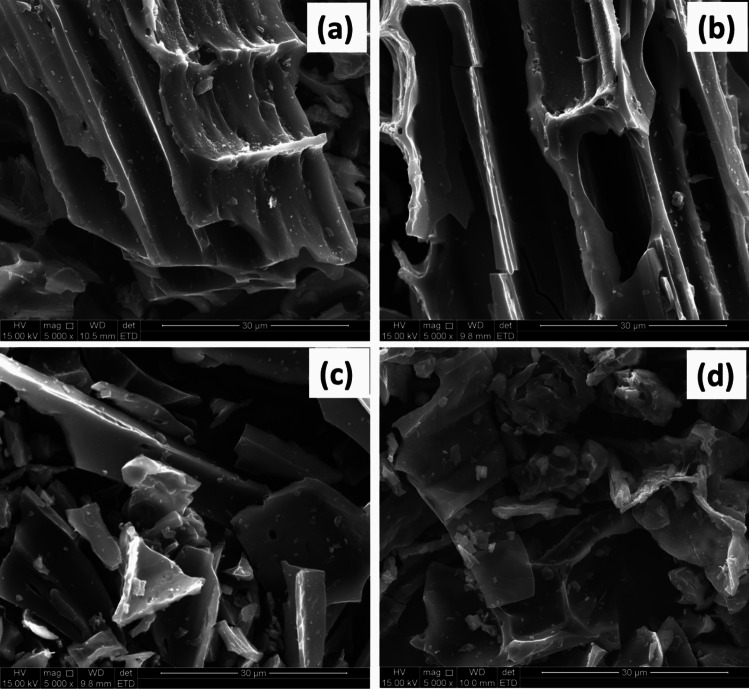
The SEM micrographs of **a** FHB and **b** FHB-SMX and **c** FBZC and **d** FBZC-SMX

SEM analysis was used to determine the microstructure of the spent and pristine adsorbents. Fig 1 displays the SEM micrographs of FBZC, FBZC-SMX, FHB, and FHB-SMX. Similar morphologies were noted in Zhang and coauthor’s most recent investigation (Zhang et al. 2020a, b). The resulting SEM micrograph (FHB) demonstrated that the pyrolysis treatment causes a large number of microscopic pores on the surface of the biochar (FHB) (see Fig. 1a). The nanocomposite exhibited structures that are amorphous and fractured with several closed pores. However, the SEM micrographs of FHB-SMX and FBZC-SMX showed few or no pores (see Fig. 1b and d). It is anticipated that the development and dispersion of the pores throughout the surface of FBZC and FHB will enhance their adsorption characteristics.

**Fig. 2:**
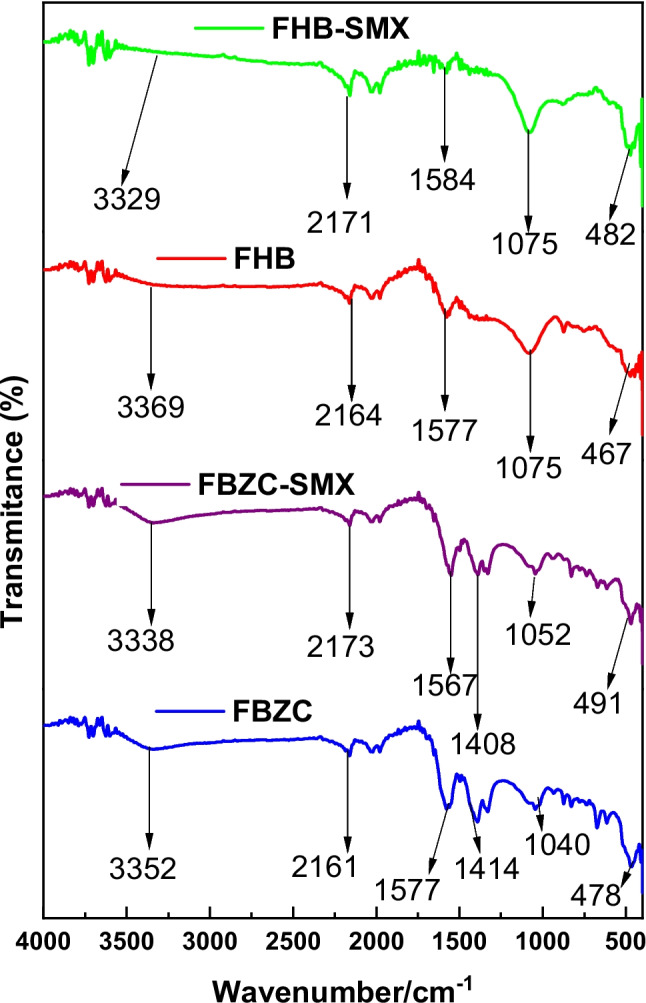
FTIR spectra of FHB, FHB-SMX, FBZC, and FBZC-SMX

The resultant nanocomposite (FBZC) and the pristine biochar (FBH) both had carbonaceous characteristic bands in their FTIR spectra (shown in Fig. 2), suggesting the predominance of carbon derivative in the material’s composition. Nonetheless, the increase in the intensity and formation of new functional groups was observed in the nanocomposite’s spectra, indicating the incorporation of ZnONPs. Both the FBH-SMX and FBZC-SMX spectra showed a varied red and blue shift in their bands following SMX adsorption, indicating the presence of active sites for SMX adsorption on the surface of FBH and FBZC. Furthermore, FBH and FBZC spectra (see Table 3) showed bands associated with silica and this could attributed to trapped oxides of silica within the husk of *Funtumia elastica* (biomass) that was employed for biochar fabrication.

**Table 3:** The observed FTIR Spectral bands (cm^−1^) and assignments

FHB	FHB-SMX	FBZC	FBZC-SMX	Assignments
3369	3329	3352	3338	υ(O–H, N–H)
2164	2171	2161	2173	υ(C-H)
1577	1584	1577	1567	υ(C = C, COO^−^,C = N)
-	-	− 1414	1408	υ_bend_(O–H)
1075	1075	1040	1052	υ_bend_(C-O)
467	482	478	491	υ_bend_(Si–O-Si, ZnO)

**Fig. 3:**
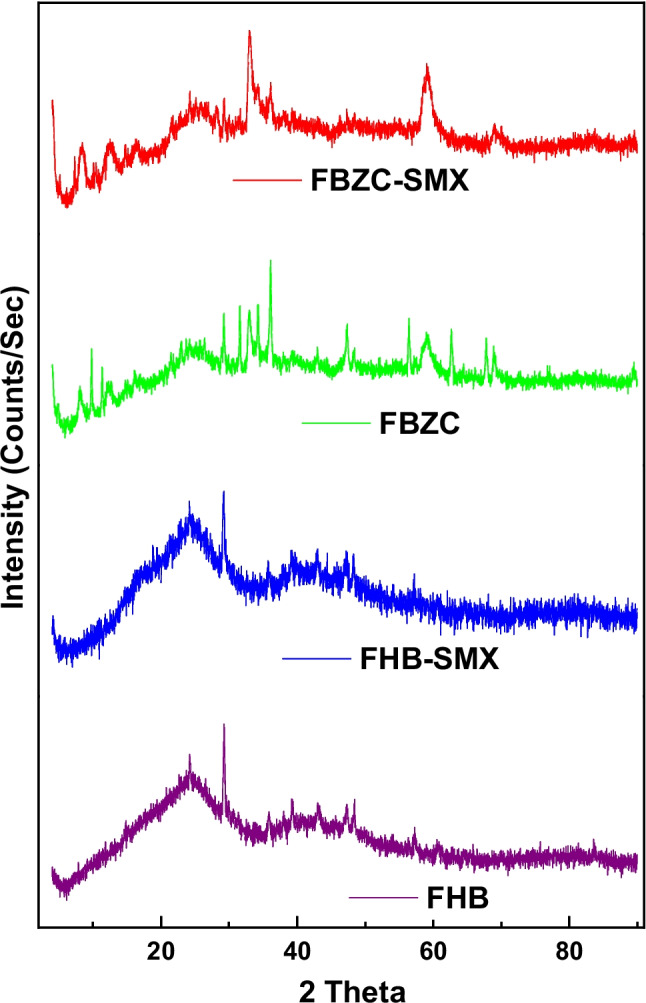
XRD pattern of FHB, FHB-SMX, FBZC, and FBZC-SMX

The X-ray diffractograms of FHB and FBZC were assessed and displayed in Fig. 3. In the diffraction pattern of FHB, the large peaks in the range of 20–30° and 40–50° are attributed to the amorphous phase of carbon (Moseenkov et al. 2023). The X-ray diffractograms pattern of FBZC composite reveals several characteristic peaks of ZnONPs which can be indexed to reflections of the ZnO wurtzite structure (JCPDS 36–1451) in addition to the observable peaks of the amorphous phase of carbon. This shows the success of the fabrication of the nanocomposite. On the other hand, similar diffraction patterns with slight shifts in the 2theta values were observed for the spent adsorbent (FHB-SMX and FBZC-SMX). This suggests that the structural composition of the adsorbent was unaltered after the adsorption step.

**Fig. 4:**
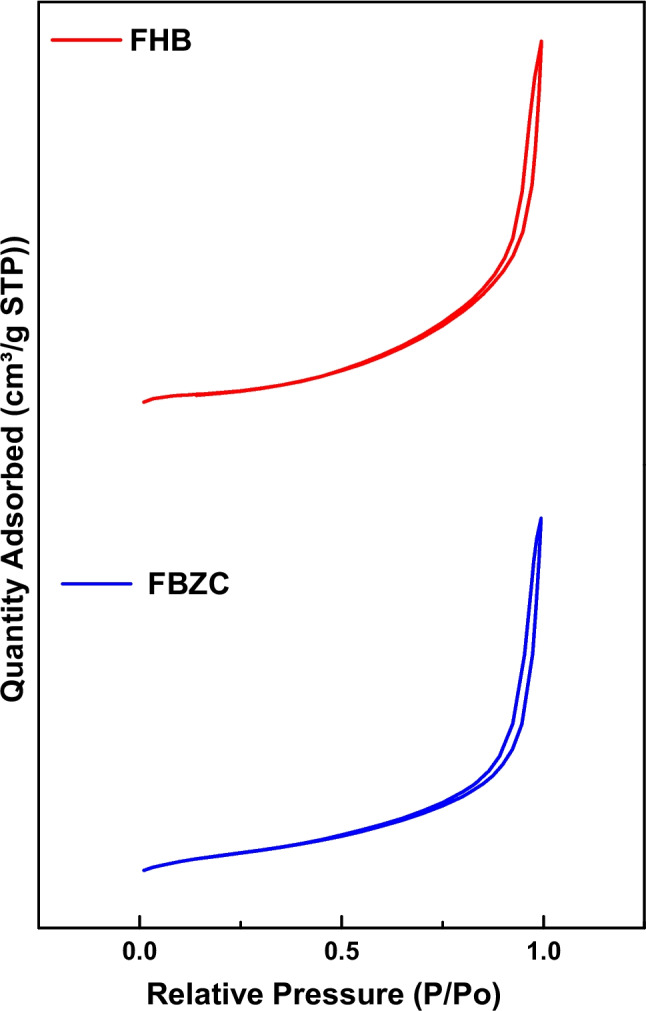
N_2_ adsorption/desorption isotherm of FHB and FBZC

Surface area, pore diameter, and pore volume are physical characteristics that can influence the potential of adsorbent to trap adsorbate. The textural properties of FHB and FBZC were estimated using the nitrogen adsorption–desorption isotherms experiment and the results are listed in Table 4. The isotherm curves obtained for FHB and FBZC followed to type III IUPAC isotherms cataloguing and had a hysteresis loop of H3 type within the relative pressure of 0.8 < P/P0 < 1 (Mukhtar et al. 2020). This demonstrates capillary condensation, suggesting a weak adsorbent-adsorbate interaction, and the predominance of a mesoporous structure (see Fig. 4).

**Table 4:** Textural properties of adsorbents

Adsorbents	Surface area/m^2^ g^−1^	Pore volume/cm^3^ g^−1^	Pore diameter/nm	pH_PZC_
FHB	0.5643	0.004054	6.2245	5.57
FBZC	1.2267	0.007049	5.6593	6.83

### Effect of initial pH

**Fig. 5:**
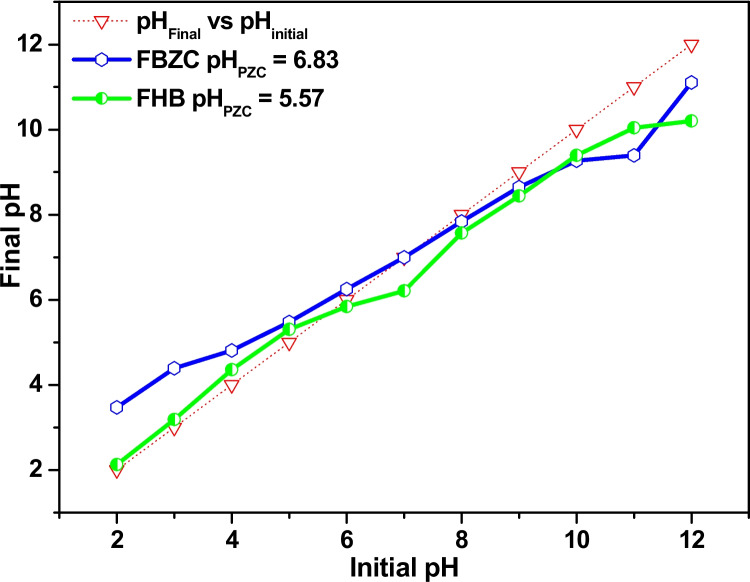
pH_PZC_ plots of FBZC and FHB

**Fig. 6:**
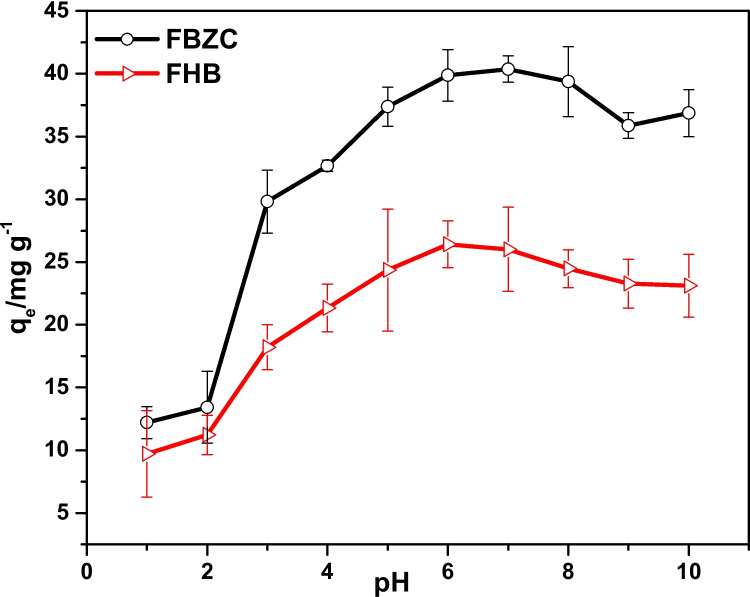
The influence of solution pH on the ability of FHB and FBZC to sequester SMX

To investigate the impact of pH on SMX uptake into the surface of FBZC and FHB, batch sorption tests were conducted within the pH range of 1 to 10 (refer to Fig. 6). The absorption of sulfamethoxazole onto FBZC and FHB showed a clear pH dependence. The findings showed that varying pH could have an impact on FBZC and FHB’s ability to adsorptively remove SMX. With an increase in the solution pH from 1 to 6, it was observed that the absorption capacity of FBZC and FHB marginally increased, but when pH increased from 7 to 10, it decreased. In the meantime, the pH_PZC_ plots of FHB and FBZC demonstrated that the net charge on the surfaces of FHB and FBZC will be zero at solution pH 5.57 and 6.83 respectively (see Fig. 5). Therefore, the surface charges of FBZC and FHB will be negatively charged above and positively charged below these pH values (FBZC = 6.83 and FHB = 5.57), respectively. However, Similar studies from different authors reported the implication of pH on SMX speciation. At pH < 1.7(pKa1) and pH > 5.7(pKa2), SMX will be SMX^+^ and SMX^−^, respectively (Zhao, Zhao, et al. 2022a, b). The variation in the net charge of the SMX molecule is attributed to the protonation of an amino group and the deprotonation of the sulfonamide group. Hence, the anionic state of the adsorbate will be dominant at solution pH 6; thus, FBZC may trap SMX via electrostatic interaction along with other forms of interactions. It is evident that ionic interactions may not play a dominant role in the uptake of SMX by FHB, rather π-π electron-donor–acceptor (EDA), pore filling, and H-bonding may play a significant role in the elimination of SMX (Fig. 6). Meanwhile, reports from several authors are consistent with our findings (Rostamian and Behnejad 2016; Zhang et al. 2020a, b; Sun et al. 2022).

### Influence of dosage on SMX adsorption

**Fig. 7:**
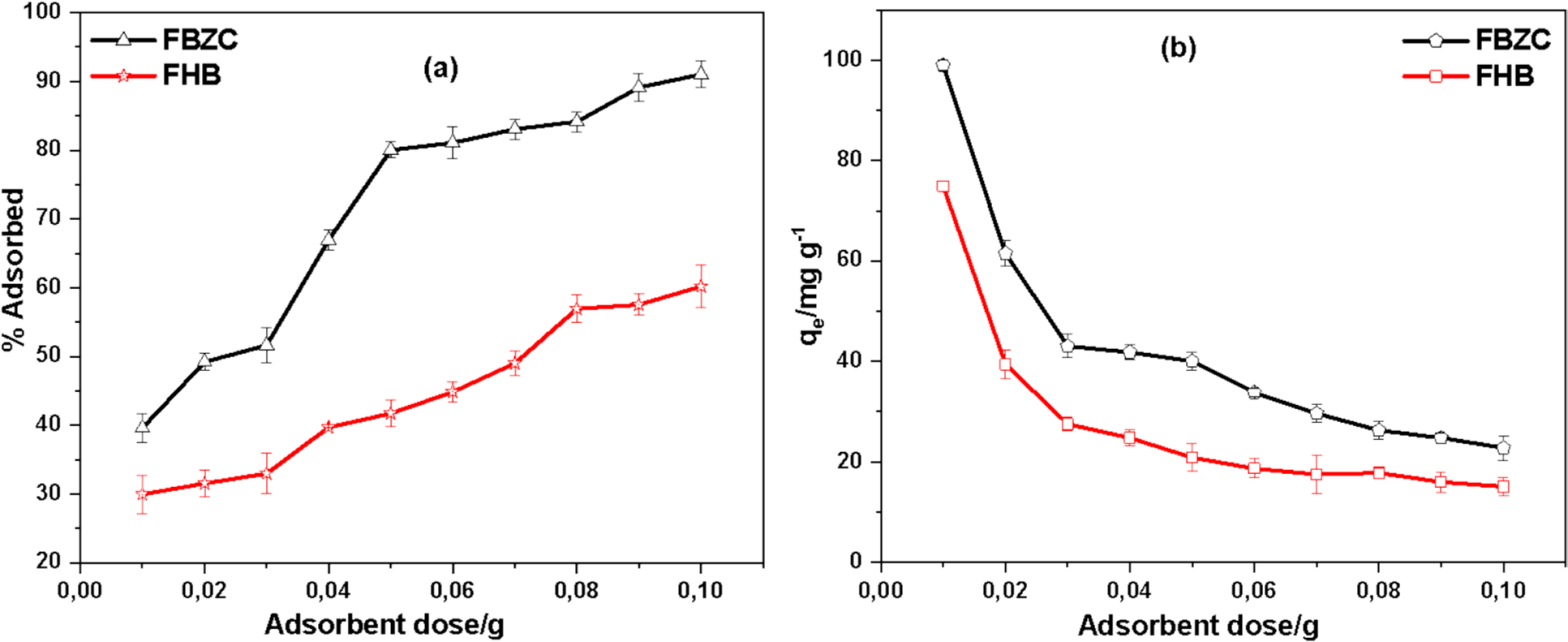
The effect of adsorbent dosage on the sequestration SXM by FBZC and FHB **a** % adsorbed and **b** adsorption capacity

Figure 7 illustrates the implication of adsorbent dose on the uptake of SMX onto the surface of FBZC and FHB at room temperature. The study demonstrated that the increase in dosage of FBZC or FHB from 0.01 to 0.1 g increased the removal efficiency of FHB from 29.16 to 57.97% and from 39.52 to 90.78% for FBZC. This phenomenon may be described by the accessibility of higher adsorption sites with fixed sorbate concentration, but further increasing the adsorbent dose results in to overlap of the available adsorption sites, thereby reducing the effectiveness of the adsorbents to sequester adsorbate (Amaku et al. 2021). However, when the dosage increased, the FBZC and FHB’s uptake capacity declined. This was likely caused by the agglomeration of the adsorbents, which might have blocked some of the sites and reduced the FBZC and FHB's uptake capacity.

### Effect of contact time

**Fig. 8:**
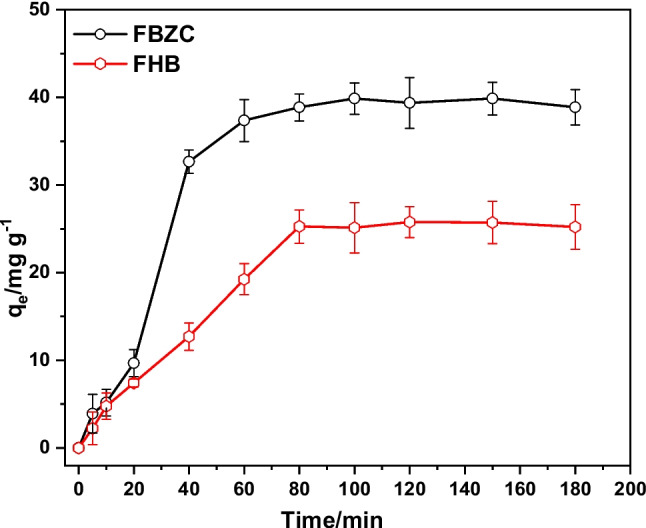
Effect of contact time on the sequestration of SMX by FBZC and FHB

**Fig. 9:**
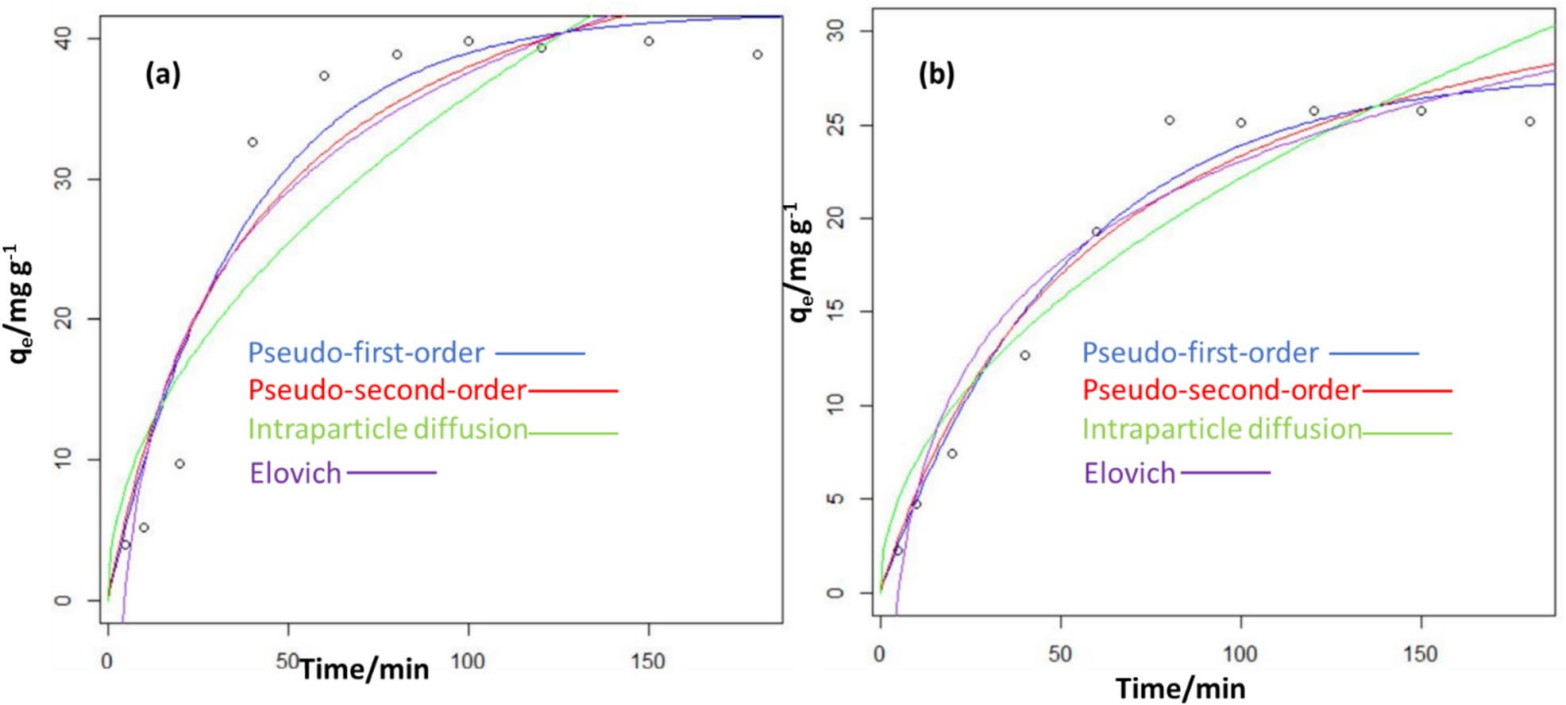
Plots of the kinetics model for the absorption of SMX onto **a** FBZC and **b** FHB

To have a thorough grasp of the chemical pathways and the SMX absorption rate, the adsorption kinetics investigation was looked into. Fig 8 illustrates how contact time affects SMX adsorption onto FBZC and FHB. After 80 min, the removal capacities of FHB and FBZC approach equilibrium. The capacities of the adsorbents were noticed to increase over time. But FBZC removes SMX more quickly than FHB does. This could be because of the ZnONPs that were added to FBZC during the fabrication process, which altered the surface chemistry of both materials. Three stages of absorption are identified: fast early adsorption, plodding adsorption, and finally no discernible uptake. The fact that adsorption equilibrium was reached in full in less than 80 min suggests that SMX molecules diffused quickly from the liquid phase to the surfaces of FBZC and FHB. As seen in Fig. 9, four kinetic models were used to fit time-dependent experimental data. It is evident from Table 3 that the Elovich and pseudo-first-order models, respectively, most accurately describe the sorption kinetics of FHB and FBZC. Therefore, Elovich kinetic models provide the best description of SMX uptake by FBZC, suggesting that SMX was adsorbed onto the FBZC via a heterogeneous or multi-mechanism process. Conversely, pseudo-first order best describes the uptake of SMX onto FHB (Table 5). The findings of this investigation are consistent with the reports from different researchers (Minaei et al. 2023; Cheng et al. 2021; Wang et al. 2020).

**Table 5:** The estimated kinetic parameters for the uptake of SMX onto FBZC and FHB

Model	Parameter	FHB	FBZC
Experimental	*q*_*exp*_/mg g^−1^	26.21	46.25
Pseudo-first order	*K*_1_/min^−1^	0.01932	0.02675
	*q*_*eq*_/mg g^−1^	27.946	41.867
	SSR	24.44	138.1
	RSE	5.45 × 10^−6^	4.154
Pseudo-second order	*K*_2_/g mg^−1^ min^−1^	4.48 × 10^−4^	4.62 × 10^−4^
	*q*_*eq*_/mg g^−1^	3.730e + 01	53.39
	SSR	37.73	207
	RSE	2.172	5.086
Intraparticle diffusion	*K*_*id*_/mg g^−1^ min^−0.5^	2.218	3.597
	*l*/mg g^−1^	1.35	2.04
	SSR	87.78	451.2
	RSE	3.123	7.08
Elovich	*α*/mg g^−1^ min^−1^	− 12.543	− 18.67
	*β*/g mg^−1^	7.732	12.22
	SSR	53.88	7.328
	RES	2.595	5.41

### Removal of SMX from real wastewater

The effectiveness of an adsorbent is observed in its ability to craftily function properly in a multi-analyte system. Real wastewater contains various analytes of different chemistry. Hence, it will be ideal to validate the efficacy of an adsorbent using an actual wastewater sample, given the potential for organic or inorganic components to interfere with a target pollutant in wastewater. Effluents were collected from the Umgeni River in KwaZulu-Natal province, South Africa ((Blue Lagoon (BLN), the confluence of the Umgeni and Msunduzi rivers (MUR), Ethekwini inlet wastewater treatment plant (WEI) and Ethekwini wastewater treatment plant (WEO)). The first step involved determining the initial concentration of SMX in the water samples. The results obtained revealed that the concentration of SMX was 0.56 mg dm^−3^ for WEO, 0.86 mg dm^−3^ for MUR, 0.75 mg dm^−3^ for WEI, and 0.68 mg dm^−3^ for BLN. A preliminary adsorption test, however, revealed 100% SMX elimination. Hence, the water samples were spiked to a concentration of 20.7 mg dm^−3^ for WEI, 30.50 mg dm^−3^ for MUR, 40.50 mg dm^−3^ for WEO, and 50.46 mg dm^−3^ BLN. However, 0.05 g of FBZC was added to 25 cm^3^ of wastewater in a 100 cm^3^ stoppered amber glass bottle; the mixture was shaken at 298 K and 120 rpm for 180 min. The concentrations of SMX decreased from 20.7 to 2.54 mg dm^−3^ for WEI, 30.5 to 1.93 mg dm^−3^ for MUR, 40.50 to 3.2 mg dm^−3^ for WEO, and 50.46 to 2.5 mg dm^−3^ BLN, where the removal percentages were 87.73% for WEI, 93.67% for MUR, 62.16% for WEO, and 95.05% for BLN, respectively. Ultimately, our findings showed that SMX can be effectively removed from wastewater using FBZC, even in the presence of components that could interfere with its removal.

### Impact of temperature of the solution and adsorbate concentration

**Fig. 10:**
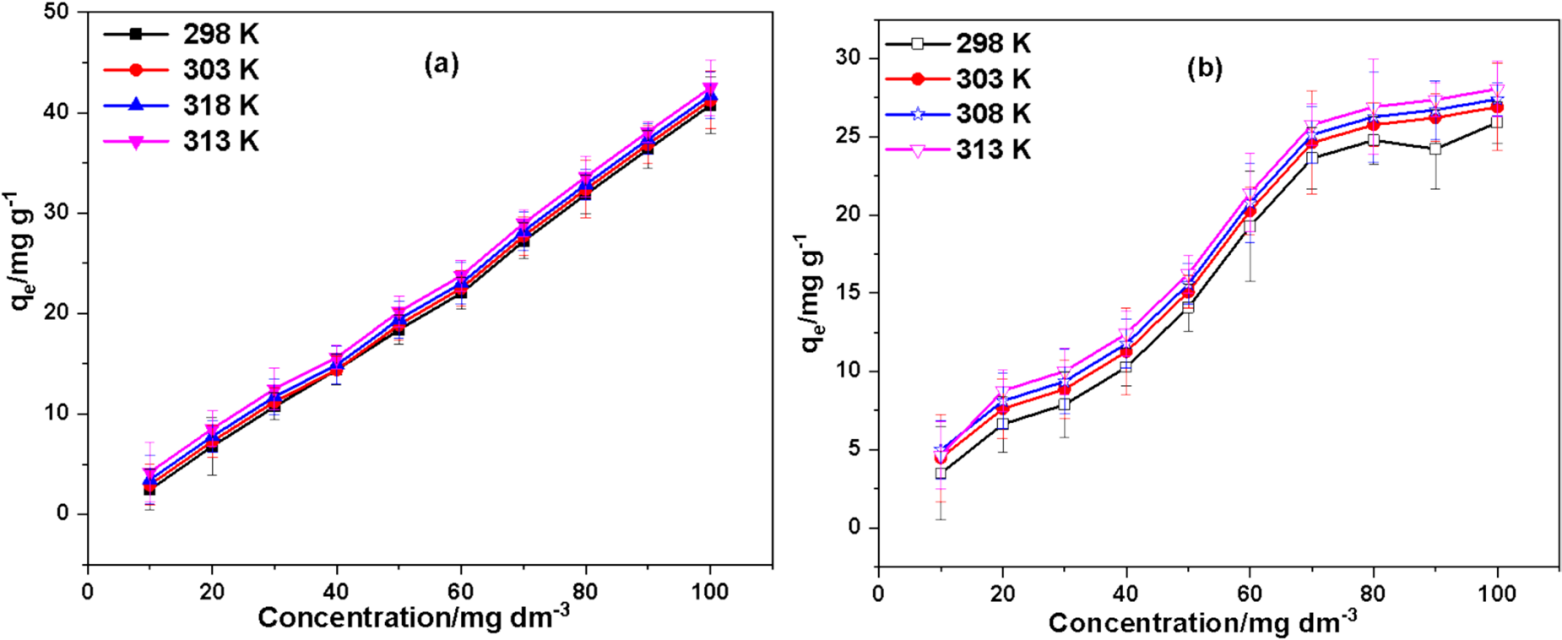
The influence of adsorbate concentration on the uptake of SMX by **a** FBZC and **b** FHB

The Initial concentration and solution temperature of the adsorbate had a substantial impact on the capacity of FBZC and FHB to sequester SMX within the temperatures between 298 and 318 K. Fig 10 demonstrates how these parameters influence the adsorption of SMX onto FBZC and FHB. It was uncovered that when the initial concentration of SMX and solution temperature increased, so did the absorption potential of FBZC and FHB. The increased mobility of the SMX brought on by the temperature rise and the waning of the retarding forces acting on the diffusing molecules may be responsible for this higher adsorption capability. Consequently, the concentration gradient enhanced the driving force responsible for the mass transfer and raised the adsorption capacity of FHB and FBZC.

### Adsorption isotherms

Isotherms plots for the uptake of SMX onto FBZC and FHB demonstrated how SMX interacted with both adsorbents (FBZC and FHB) as well as the correlation between the sorbate-sorbent ratio and its equilibrium concentration (Ce) in solution. The data analysis employed two commonly utilized isotherm models, namely the Freundlich and Langmuir models. According to the Langmuir model, monolayer adsorption accounts for the adsorbate molecules’ adsorption on a homogenous surface in the absence of any interactions between the adsorbed molecules (Nguyen and Do 2000). Due to the presence of distinct functional groups on heterogeneous surfaces, the Freundlich isotherm model is widely used in a multi-layer sorption process. It includes a variety of adsorbent-adsorbate interactions to characterize non-ideal and reversible adsorption (Freundlich 1906). Table 6 shows the estimated parameters for various isotherms models employed in this study. The SSR value indicates that Freundlich provides the best description of SMX absorption by FBZC. The finding reveals the heterogeneous surface of FBZC and the multi-layer coverage of SMX. When *n* is more than 1, it indicates that the FBZC has auspiciously absorbed SMX. Similar results were also reported by different authors (Yao et al. 2012). Furthermore, the SSR values suggested that the Langmuir isotherm best fitted the equilibrium data for the uptake of SMX by FHB. The non-linear Langmuir isotherm model was employed to estimate the monolayer uptake capacity (*q*_*max*_) value for FBZC, which was 61.91 mg g^−1^. This value was significantly greater than the highest adsorption capacity of the majority of published adsorbents (see Table 7).

**Table 6:** Adsorption isotherm model parameters for SMX sequestration by FBZC and FHB

**Adsorbent**	**Isotherm**	**Parameters**		**Temperature**		
			**298 K**	**303 K**	**308 K**	**318 K**
**FHB**	Langmuir	*qm*	59.34705	52.49019	49.8822	45.281
		*b*	0.017875	0.026382	0.03148	0.04299
		SSR	117.936	121.837	137.5944	131.625
		RSE	3.84	3.903	4.147	4.056
	Freundlich	*K*_*F*_	1.864750	2.921462	3.590854	4.509355
		*n*	1.426227	1.659306	1.803092	1.986764
		SSR	128.41	124.15	139.459	121.079
		RSE	4.007	3.939	4.175	3.890
**FBZC**	Langmuir	*qm*	56.354	58.1763	60.2578	61.9082
		*b*	0.014352	0.01078	0.02357	0.01889
		SSR	1889	1642	1664	1714
		RSE	3.151	4.087	3.732	3.078
	Freundlich	*K*_*F*_	0.112157	0.16719	0.35882	1.03260
		*n*	0.49864	0.52369	0.59603	0.74282
		SSR	75.801	88.3568	97.854	120.369
		RSE	3.078	3.323	3.497	3.879

*SSR*, sum of squared residuals; *RSE*, the residual squared errors

**Table 7:** Comparison of different sorbents’ Langmuir maximum adsorption potential with the capacity of FBZC and FHB to sequester SMX

Adsorbents	pH	Temperature (℃)	*q*_*max*_	Reference
MIP-MBC	7–9	25	16.22	Li et al. (2023)
Ni@CNFs	7.0	25	22.7	Lan et al. (2016)
Carbon nanotube	8.0	-	3.07	Liu et al. (2017)
nHAP@biochar	~ 6	25	14.52	Li et al. (2020)
DCS	5.0	-	36.90	Lun et al. (2023)
Biochar/Activated carbon	8.0	25	1.16–4.88	Zhao et al. (2022a, b)
Activated carbon	5–7	25	16.15	Liu et al. (2019)
Biochar	6.5	25	19.09	Reguyal and Sarmah (2018a)
BCN	-	10	28.75	Sun et al. (2022)
CSAC	5.6	25	6.4	Pamphile et al. (2019)
FBH	6	318	45.281	This study
FBZC	6	318	61.91	This study

### Mechanism of adsorption

**Fig. 11:**
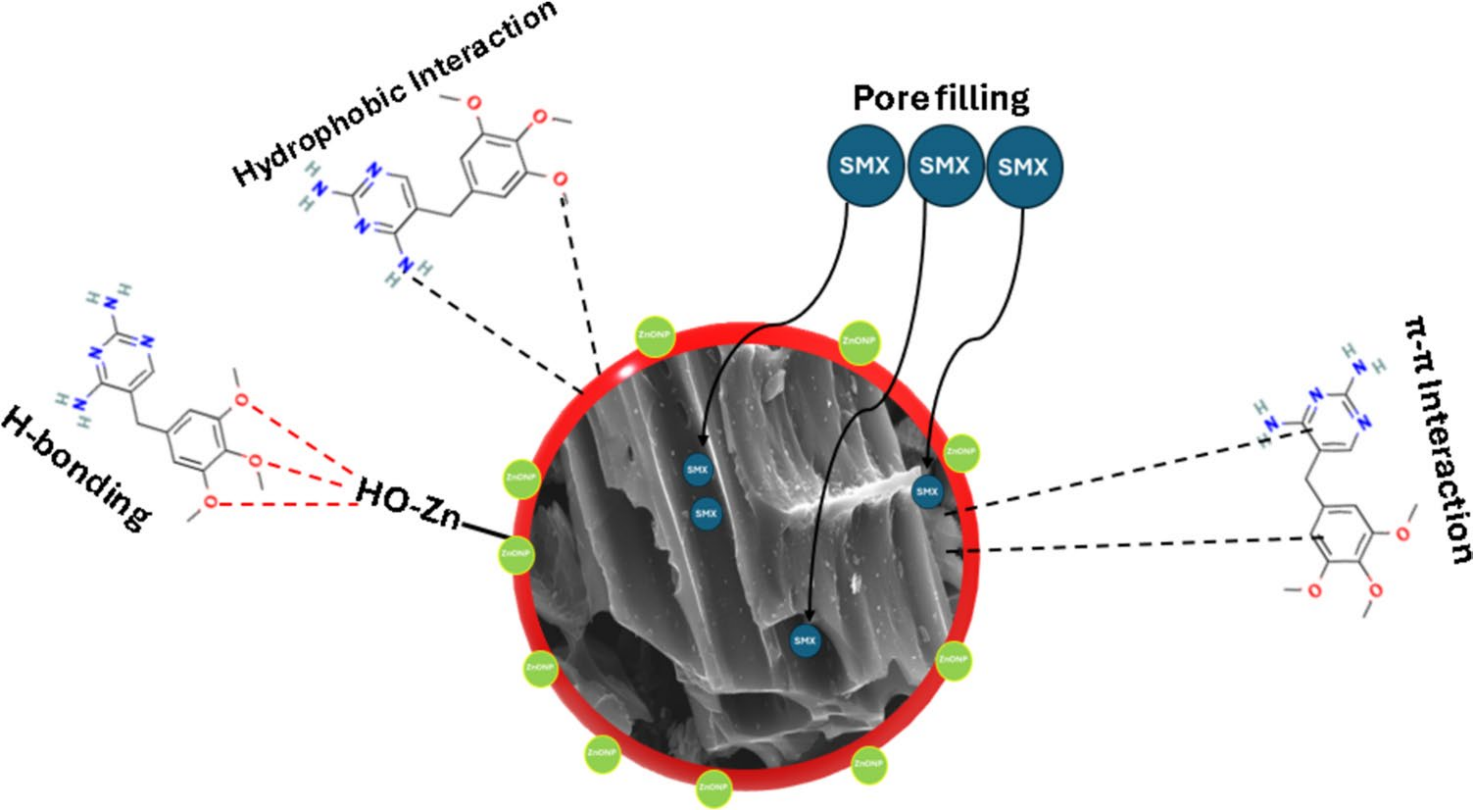
The mechanism of SMX adsorption onto FBZC

The uptake of organic molecules is generally governed by four main mechanisms, and they include hydrogen bonding, π-π bonding, and electrostatic interactions (Ngo et al. 2023). To have a comprehensive insight into the adsorptive path of SMX removal by FBZC, spectroscopic techniques such as XRD, SEM, and FTIR were used to investigate the pristine and spent adsorbents. The relationship between the surface charge and solution pH was also assessed. The observations made on the SEM micrograph suggest ease of fixation of SMX onto the surface/pores of FBZC. On the other hand, the slight increase of the surface area and pore volume of FBZC may aid pore entrapment of SMX. The study also demonstrated from the thermodynamic point of view that the adsorption of SMX onto FBZC is also feasible and spontaneous. The FTIR reveals the availability of chemical moieties (C = C, C = O, and C = N) that sustain robust π-electrons on the surface of pristine and spent adsorbents. On the other hand, the benzene rings and amine groups on SMX may accept π-electrons from the functional groups on the surface of the adsorbents resulting in π-π electron interactions (Liu et al. 2021a). This reaction path is further justified by the varied intensities and slight shifts in bands that were observed on the FTIR spectrum of FBZC-SMX, FBZC, FHB, and FHB-SM. The interaction between the donor hydrogen sites of FBZC with N and O of SMX may result in hydrogen bonds between the adsorbate and the adsorbent (Akpotu and Moodley 2018). Furthermore, SMX is known to have a pKa of 1.7 and 5.7, which implies that at pH < 1.7, SMX is largely cationic, at pH between 1.7 and 5.7, the molecule of SMX will be neutral, and at pH > 5.7, SMX will be chiefly anionic (Minaei et al. 2023). An optimum pH of 6 was obtained from the influence of the pH experiment with a pH_PZC_ of 6.83 and 5.57 for FBZC and FHB respectively. Hence, at pH 6, electrostatic interaction will be the dominant mechanism for the uptake of SMX onto FBZC in addition to the aforementioned mechanisms. This justifies the superior capacity of FBZC over FHB (see Fig. 11). Similarly, various authors have reported a multi-path removal mechanism for the sequestration of SMX from the aqueous phase (Pamphile et al. 2019; Jawad et al. 2021).

### Thermodynamic study

The values of the estimated thermodynamic variables for the absorption of SMX onto FBZC and FHB are shown in Table 8. As the solution temperature rises, the adsorption process becomes more efficient and useful. The parameters (enthalpy change (ΔH°), entropy change (ΔS°), and change in Gibbs energy (ΔG°)) that are associated with thermodynamics were estimated using Eqs. 4 and 5 (Yurtay and Kılıç 2023).

**Table 8:** Estimated thermodynamic parameters for the adsorption of SMX onto FHB and FBZC

Adsorbents	T/K	ΔG°/kJ mol^−1^	ΔH/ kJ mol^−1^	ΔS/J K^−1^ mol^−1^
	298	− 21.0881	2.58	6.73
FBZC	303	− 20.1736		
	308	− 20.7003		
	318	− 21.4311		
FHB	298	− 17.088		
	303	− 18.2228	20.04	15.16
	308	− 18.9678		
	318	− 20.0253		

4 $$\Delta \text{G}^\circ =-RT\text{ln}K$$

Based on the linear plot’s slope and intercept of $\left(\text{ln}K\right)$ against 1/*T*, values of ΔH° and ΔS° were estimated respectively. The constant *K* can be calculated by multiplying the Langmuir constants *q*_*max*_ and 1000(b), *T* is the solution temperature (K), and *R* is 8.314 J mol^−1^ K^−1^ (Doke and Khan 2013; Milonjić 2007).

5 $$\text{ln}K=-\frac{\Delta \text{H}^\circ }{RT}+\frac{\Delta \text{S}^\circ }{R}$$

The adsorption process is endothermic when the value of ∆H° is positive, and exothermic when it is negative. Conversely, the increased and decreased unpredictability of the adsorptive process was shown by the positive and negative values of ∆S°, respectively. Hence, amplified randomness at the solid–liquid boundary was observed for the uptake of SMX onto FBZC and FHB. The favourable adsorption and spontaneity of the removal process were apparent in the negative ∆G° values (see Table 8). Comparable findings were also seen in the adsorption of SMX by chemically modified biochar (Minaei et al. 2023).

### Antioxidant activities

The antioxidant characteristics of FHB and FBZC were assessed using the 2,2–diphenyl–1–picrylhydrazyl hydrate (DPPH) free radical scavenging method. Table 9 demonstrates that inhibitory activity FHB and FBZC were dependent on the concentration of the agents (FHB and FBZC) employed. On the other hand, ZnONP-modified biochar (FBZC) exhibited higher inhibition than FHB. This phenomenon could be attributed to the higher face area to volume ratio of the composite as observed from the textural properties of adsorbents (see Table 4). It may also be associated with the propagation of electron density out of the oxygen of the nanocomposite to nitrogen-containing lone pair electrons in DPPH. Several authors have demonstrated the synergistic implication of incorporating ZnONPs into nanocomposite fabrication and in most cases, the effect is observed in the antioxidant activities of the agent (Hosny et al. 2022; Kamal et al. 2022).

**Table 9:** Antioxidant activity of FHB and FBZC

Concentration (µg cm^−3^)	Percentage inhibition (%)		
	FBZC	FHB	Ascorbic acid
10	5.33	2.28	91.09
30	7.87	3.97	92.27
60	9.35	4.91	94.37
120	10.56	5.26	94.53
240	15.84	6.93	95.18

### Desorption and regeneration studies

**Fig. 12:**
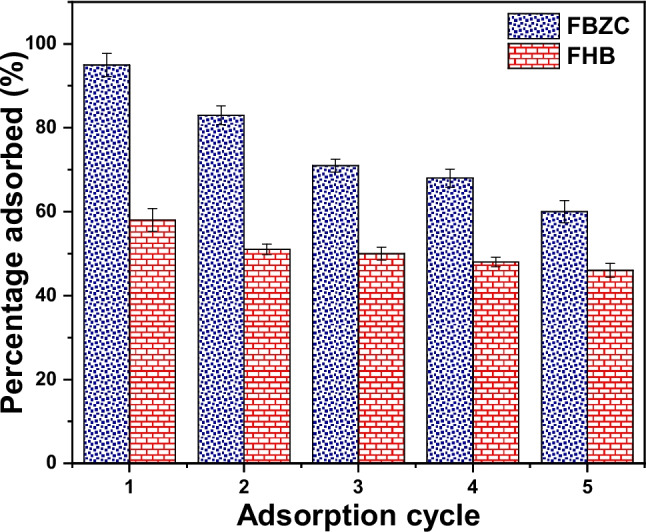
Reusability FHB and FBZC for the uptake of SMX after ethanol washing (100 cm^3^ of a 100 mg dm.^−3^ of SMX solution adjusted to pH 6 and 30 mg of FHB or FBZC at 298 K)

Adsorbent regeneration is crucial because it demonstrates how much of the adsorbent material may be reused. The significant drop in expenses related to creating new adsorbents and the decrease in secondary pollutants as a result of disposing of used adsorbents demonstrate the adsorbent’s economic and environmental feasibility. The effectiveness of four solvents was investigated for SMX desorption. Preliminary assessments of deionized water, NaOH, acetone, and ethanol were performed to determine which eluent is best for SMX desorption. Deionized water, NaOH, acetone, and ethanol demonstrated 3.63%, 76.22%, 82.64%, and 96.17% regeneration efficiency respectively. Hence, ethanol was used for the adsorption–desorption of SMX onto FHB and FBZC. It was noticed that the eluting efficiency of ethanol declined after the second cycle; however, the third-fifth circle maintained relatively same desorption efficiency. Following five regeneration cycles, the effectiveness of the adsorption of FHB and FBZC was noticed to decline from 95.84 to 62.75% and from 58.25 to 46.89% respectively (see Fig. 12). FBZC exhibited high uptake capacity and easy regeneration, It would considerably lower the total cost of using them as adsorbents and turn them into valuable resources for environmental remediation practices.

### Antibacterial activity assay

The bacterial susceptibility to the water treatment agent (FHB and FBZC) was investigated using the diffusion-base technique. The technique employs the zone of inhibition, which is proportional to the degree of bacterial susceptibility to FHB or FBZC. The amount of antibacterial activity is defined by the zone of inhibition surrounding the impregnated disc, which has a zone diameter of millimeters. Gram-negative (*Escherichia coli*) and gram-positive (*Staphylococcus aureus*) organisms were employed to assess the inhibitory activity of FHB and FBZC. It was observed that the more active the water treatment agents are, the wider the zone of inhibition. The results were read after 24 h of incubation at 37 ℃ using a concentration of 250 µg (see Figs. 1S and 2S). FHB and FBZC demonstrated NIL and a 15.2-mm inhibition zone against *S. aureus*. In a similar trend, FHB and FBZC exhibited zones of inhibition of NIL and 15.0 mm against *E. coli* (see Table 10)*.* Hence, the nanocomposite has demonstrated good antimicrobial activity against the investigated microbial strains. The synergistic activity between ZnONPs and the biochar was most observed with the gram-positive organism. The mechanism responsible for the antimicrobial activity of FBZC could be associated with the ease in dislodging the ZnONPs from its support onto the membrane of the cells, which in turn mitigates the integrity of the cell wall. After the perforation of the cell wall, the zinc ions will bind the proteins and enzymes inside the cell, which in turn will disrupt vital cellular processes that may result in cell death [80]. Several reports have demonstrated the benefits of the composite embedded with nanoparticles for innovative applications (Alkasir et al. 2020; Chiriac et al. 2022; Râpă et al. 2021).

**Table 10:** The inhibitory effect of antimicrobial agents (FHB and FBZC) on *Staphylococcus aureus and Escherichia coli*

Samples	Zone of inhibition (mm)	
	*E. coli*	*S. aureus*
FBZC	15.0	15.2
FHB	NIL	NIL
STD	35.0	37.2

## Conclusions

A newly fabricated biochar (FHB) prepared from *Funtumia elastica* husk was further modified with ZnONPs and ascorbic acid to obtain a potent water treatment agent (FBZC) with antimicrobial and antioxidant characteristics. FHB and FBZC were assessed for their capacity to eliminate SMX from wastewater. FBZC demonstrated robust surface morphology and essential chemical moieties at its surface. Meanwhile, ideal conditions for efficient sequestration of SMX by FHB and FBZC were established to be 180 min agitation time, solution pH of 6 at 318 K solution temperature. Under these conditions, FBZC and FHB had a maximum monolayer potential (*q*_*max*_) of 61.91 mg g^−1^ and 45.281 mg g^−1^ respectively. Additionally, the Freundlich and Langmuir models, respectively, better described the experimental isotherm data acquired for the absorption of SMX onto FBZC and FHB respectively. Elovich and pseudo-first-order kinetic models provided the best description of the time-dependent adsorption data of FBZC and FHB. Owing to the predicted thermodynamic characteristics, FBZC and FHB’s adsorption of SMX was endothermic, spontaneous, and entropy-driven. Consequently, our work offers a unique water treatment agent that can clean and disinfect wastewater containing sulfamethoxazole. Hence, future study will focus on pilot scale study for the application of FBZC in the continuous flow wastewater treatment technique.

## Supplementary information

Below is the link to the electronic supplementary material.

Supplementary file1 (DOCX 2198 KB)

## Data Availability

Not applicable.
