# Supplementary material for: Functional ZnONPs‐modified biochar derived from Funtumia elastica husk as an efficient adsorbent for the removal of sulfamethoxazole from wastewater
Source: Environ Sci Pollut Res Int. 2024 Nov 25;31(57):65496–512. doi: 10.1007/s11356-024-35594-8 (PMC11632023; doi:10.1007/s11356-024-35594-8)
Supplement: Supplementary file 1 — Supplementary file1 (DOCX 2198 KB) [file 11356_2024_35594_MOESM1_ESM.docx]

**Functional ZnONPs‐modified biochar derived from *Funtumia Elastica* husk as an efficient adsorbent for the removal of Sulfamethoxazole from wastewater**

**^*^****James Friday Amaku^1,2^ · Fanyana M. Mtunzi^1^**

^1^Wastewater Treatment Research Laboratory, Department of Biotechnology and Chemistry, Vaal University of Technology, Vanderbijlpark 1911, Gauteng, South Africa

^2^Department of Chemistry, Michael Okpara University of Agriculture Umudike, P.M.B 7267 Umuahia, Abia State, Nigeria

*Corresponding author: fridaya@vut.ac.za


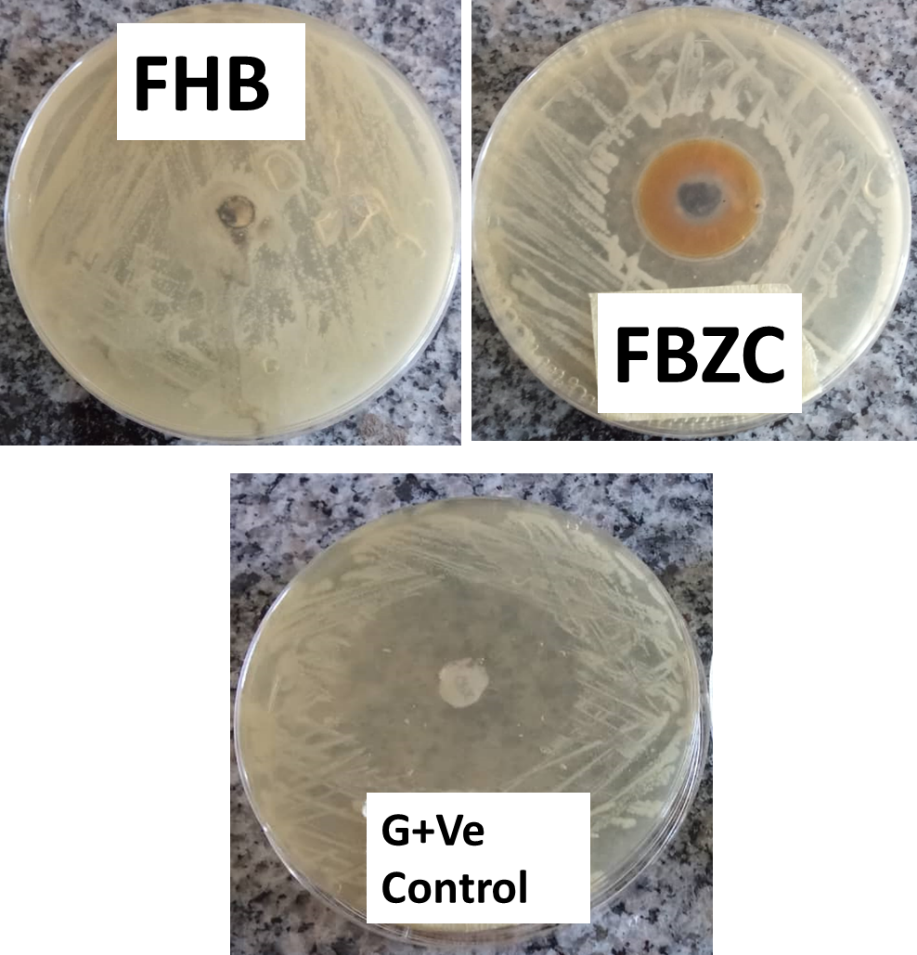


**Fig 1S:** Antibacterial activity of FHB and FBZC for *S. aureus.*


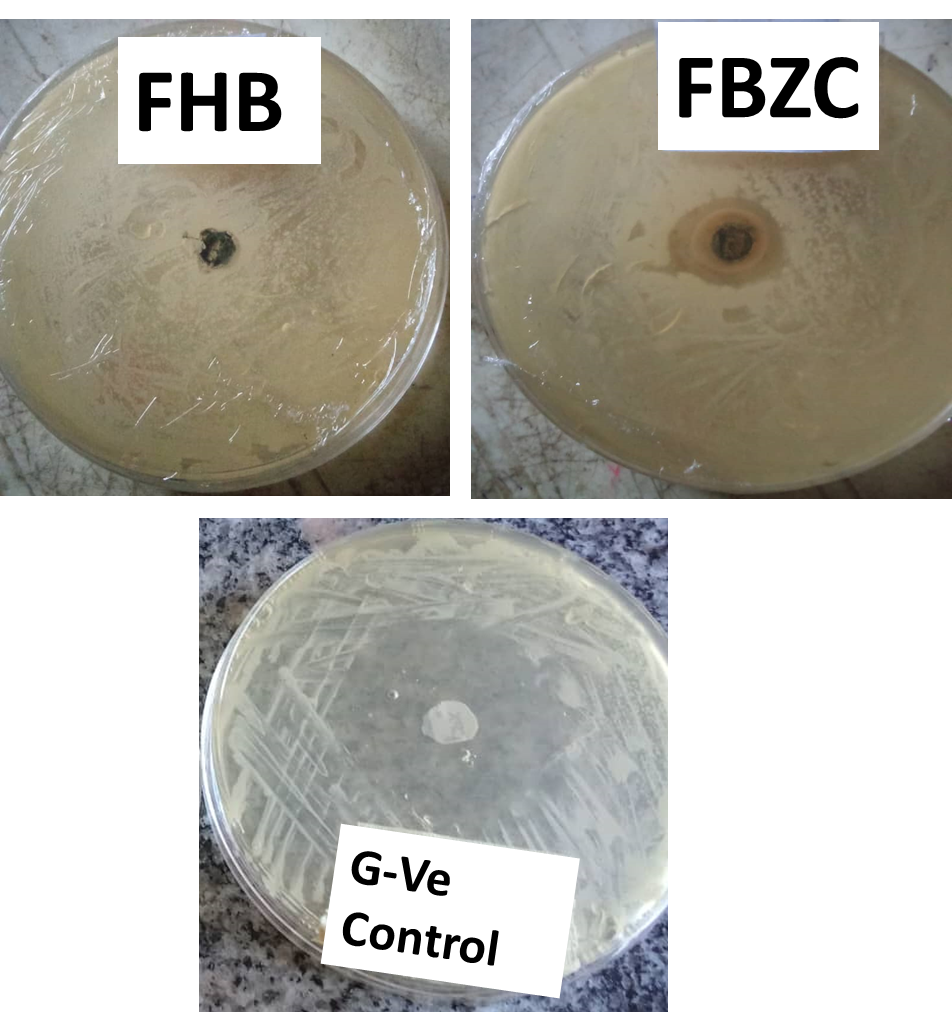


**Fig 2S:** Antibacterial activity of *E. coli* for FHB and FBZC
